# Geriatric nutritional risk index predicts the prognosis of gastric cancer patients treated with immune checkpoint inhibitors

**DOI:** 10.1097/MD.0000000000037863

**Published:** 2024-04-26

**Authors:** Bao Liu, Limin Zhang

**Affiliations:** a Harbin Medical University Cancer Hospital, Harbin Medical University, Harbin, China.

**Keywords:** gastric cancer, geriatric nutritional risk index, immune checkpoint inhibitors, nutritional status, prognostic factor

## Abstract

The nutritional status is closely linked to the immune function of patients. Previous studies have demonstrated the utility of the Geriatric Nutritional Risk Index (GNRI) in assessing nutritional status. The aim of this study is to investigate the prognostic significance of GNRI in patients with gastric cancer who received immune checkpoint inhibitor (ICI) therapy. The study enrolled 89 gastric cancer patients who received different types of immune checkpoint inhibitors (ICIs) between August 2016 and December 2020, along with 57 patients who underwent chemotherapy during the same period as a control group. The GNRI cutoff point was established based on prior research. Differences in clinical and pathological features were analyzed using the Chi-square test or independent samples t-test. Univariate and multivariate analyses were used to identify prognostic factors for both progression-free survival (PFS) and overall survival (OS). Furthermore, nomograms were created to predict the likelihood of patient survival. There were 31 cases (21.2%) with GNRI < 92.00 and 115 cases (78.8%) with GNRI ≥ 92.00. Patients with low GNRI had significantly shorter PFS (21.33 months vs 28.37 months, *P* = .001) and OS (33.06 months vs 41.63 months, *P* = .001) than those with high GNRI, among all patients. Similar results were also found in patients treated with ICIs. Additionally, GNRI was identified as an independent prognostic factor. The C-index and 95% CI of the nomograms for predicting survival probabilities were 0.667 (0.600–0.735) and 0.685 (0.622–0.749), respectively. GNRI was significantly associated with survival time in patients with gastric cancer who received ICIs, patients with low GNRI had shorter PFS and OS. GNRI might be able to identify patients who might benefit from ICIs.

## 1. Introduction

While there has been a decline in morbidity associated with gastric cancer in recent years, it remains the fifth most diagnosed cancer, particularly in Asian countries like Japan, Korea, and China.^[1,2]^ With the advent of various treatment options, such as surgery, adjuvant therapy, targeted therapy, and immunotherapy, combination therapy has become the primary choice for treating gastric cancer.^[3]^ Therefore, selecting the appropriate treatment based on the disease stage and physical condition is crucial for improving patients’ survival rates.

Immune checkpoint inhibitors (ICIs) have demonstrated remarkable therapeutic benefits in various types of cancer,^[4]^ including gastric cancer, as supported by several studies.^[5,6]^ However, their use in gastric cancer presents new challenges, as only a small subset of patients benefits from ICIs.^[7]^ Therefore, there is a critical need to identify potential biomarkers that can guide treatment selection and predict patient outcomes. While current methods such as microsatellite instability (MSI) can help assess the efficacy of immunotherapy, they are often complex, expensive, and may not apply to all patients with gastric cancer.^[4,8–11]^ Furthermore, microsatellite stability and PD-L1 negativity may not always exclude the possibility of benefiting from ICIs. Malnutrition, which is directly linked to poor treatment tolerance, reduced immune function, increased postoperative complications, and tumor aggressiveness, can also predict prognosis.^[12–14]^ Nutritional status is also correlated with immune function, which is a crucial component of ICIs’ effectiveness.^[15,16]^ As such, patients’ nutritional status may affect the response to ICIs, as confirmed by some studies on nutritional indices.^[17,18]^

The Geriatric Nutritional Risk Index (GNRI), which includes changes in body weight, was established in 2005.^[19]^ Some comprehensive studies have shown that GNRI has higher prognostic prediction ability than other markers.^[20,21]^ In 2015, GNRI was first used for gastrointestinal cancer.^[22]^ Given the association between nutritional status and immune function, and the fact that nutritional status can affect the efficacy of ICIs, exploring the potential of GNRI as a noninvasive biomarker for predicting the efficacy of ICIs is of great significance.

## 2. Materials and methods

### 2.1. Patients

The study included 89 patients with gastric cancer who received various types of immune checkpoint inhibitors (ICIs) from August 2016 to December 2020. A control group of 57 patients who received chemotherapy during the same period was also included. Patients who received ICIs or chemotherapy and had complete clinical data were included in the study. Clinical data were collected from electronic medical records and analyzed in accordance with the Helsinki Declaration and its amendments. Informed consent was waived by the Ethics Committee of Harbin Medical University Cancer Hospital due to the retrospective nature of the investigation (Number: 2019-57-IIT).

### 2.2. Geriatric nutritional risk index

Since gastric cancer patients could usually measure their height normally, the measured height was directly brought into the Lorenz formula calculation in this study. GNRI was calculated as follows: GNRI = [1.489 × albumin (g/L)] + [41.7 × (weight/WLo)]. The Lorenz formula that calculated the ideal weight was as follows: male = height – 100 – [(height − 150)/4]; female = height – 100 − [(height − 150)/2.5]. The cutoff value of GNRI referred to the standards of previous studies.^[23]^ Due to the limitation of the number of cases, patients were divided into two groups (GNRI < 92.00 group and GNRI ≥ 92.00 group).

### 2.3. Data collection

All patients included in this study were followed up via telephone calls. Progression-free survival (PFS) was defined as the duration between the start of treatment and the point of disease progression, which was assessed through laboratory tests and imaging. If disease progression could not be determined, the date of death or the last follow-up was considered as the endpoint for PFS analysis. Overall survival (OS) was defined as the time between the start of treatment and death or the last follow-up.

### 2.4. Statistical analysis

In this study, continuous variables were presented as mean ± standard deviation (SD) and compared using independent samples *t* test. Categorical variables were presented as the number of patients (percentage, %) and compared using Chi-square test or Fisher’s exact test. Kaplan–Meier survival curves and Log-rank test were used to compare the differences in survival time. Univariate and multivariate analyses were performed to identify prognostic markers, and the relative risks were evaluated using the hazard ratio (HR) and 95% confidence interval (CI). Nomograms were constructed, and calibration curves were plotted to assess the predictive effectiveness of the nomograms. All calculations were performed using R 4.2.2, and a two-sided *P* value of < .05 was considered statistically significant.

## 3. Results

### 3.1. Patient characteristics

This study included a total of 146 participants, of whom 102 (69.9%) were men and 44 (30.1%) were women, with an average age of 58.05 (9.84) years. There were 31 (21.2%) cases in the GNRI < 92.00 group and 115 (78.8%) cases in the GNRI ≥ 92.00 group. Patients with GNRI < 92.00 had a lower BMI (*P* < .001) (Table 1). As the blood parameters in this study were non-normally distributed, patients were grouped according to the median of blood parameters, and the difference between the two groups was analyzed. We found that GNRI was related to γ-glutamyl transpeptidase (γ-GGT), total protein (TP), prealbumin, red blood cell (RBC), hematocrit (Hct), hemoglobin (Hb), alpha-fetoprotein, and carbohydrate antigen 125II (CA125II) (all *P* < .05). Using Fisher’s exact test, we found that patients with low GNRI had lower albumin levels (*P* < .001). The median values and detailed information of these blood parameters were presented in Table 2.

**Table 1:** The clinical information of all patients.

n	GNRI < 92.00	GNRI ≥ 92.00	*P*
	31	115	
Sex (%)			
Male	21 (67.7)	81 (70.4)	.772
Female	10 (32.3)	34 (29.6)	
Age (yr), mean (SD)	58.29 (8.85)	57.74 (10.64)	.744
BMI (kg/m^2^), mean (SD)	19.92 (2.49)	23.60 (3.19)	<.001
SLNM (%)			
Yes	5 (16.1)	13 (11.3)	.468
No	26 (83.9)	102 (88.7)	
Surgery (%)			
Yes	17 (54.8)	69 (60.0)	.604
No	14 (45.2)	46 (40.0)	
Radical resection (%)			
Yes	23 (37.1)	40 (48.8)	.162
No	39 (62.9)	42 (51.2)	
Primary tumor site			
Upper 1/3	5 (16.1)	16 (13.9)	.555
Middle 1/3	12 (38.7)	33 (28.7)	
Low 1/3	13 (41.9)	56 (48.7)	
Whole	1 (3.2)	10 (8.7)	
Borrmann type (%)	2 (6.5)	7 (6.1)	
Borrmann I + II	2 (6.5)	7 (6.1)	.859
Borrmann III + IV	15 (48.4)	60 (53.9)	
Unknown	14 (45.2)	46 (40.0)	
Tumor size (%)			
<50 mm	12 (38.7)	29 (25.2)	.273
≥50 mm	7 (22.6)	25 (21.7)	
Unknown	12 (38.7)	61 (53.0)	
Differentiation (%)			
Poor	20 (64.5)	75 (65.2)	.988
Moderately + Well	9 (29.0)	32 (27.8)	
Unknown	2 (6.5)	8 (7.0)	
Pathology (%)			
Adenocarcinoma	21 (67.7)	76 (66.1)	.445
Others*	6 (19.4)	31 (27.0)	
Unknown	4 (12.9)	8 (7.0)	
TNM stage (%)			
I	2 (3.2)	8 (9.8)	.502
II	6 (9.7)	8 (9.8)	
III	17 (27.4)	22 (25.6)	
IV	38 (59.7)	45 (54.9)	
Lauren type (%)			
Intestinal	8 (25.8)	25 (21.7)	.535
Diffuse	3 (9.7)	18 (15.7)	
Mixed	3 (9.7)	20 (17.4)	
Unknown	17 (54.8)	52 (45.2)	
PD-1 (%)			
Negative	13 (41.9)	52 (45.2)	.882
Positive	3 (9.7)	13 (11.3)	
Unknown	15 (48.4)	50 (43.5)	
PD-L1 (%)			
Negative	11 (35.5)	31 (27.0)	.301
Positive	5 (16.1)	34 (29.6)	
Unknown	15 (48.4)	50 (43.5)	
Treatment (%)			
ICIs	17 (54.8)	72 (62.6)	.431
Chemotherapy	14 (45.2)	43 (37.4)	

BMI = body mass index, ICIs = immune checkpoint inhibitors, SLNM = supraclavicular lymph node metastasis.

* Others, include mucinous carcinoma, signet ring cell carcinoma, mixed carcinoma, unknown.

**Table 2:** The blood parameters of all patients.

n	GNRI < 92.00	GNRI ≥ 92.00	*P*
	31	115	
ALT (%)			
<14.5 U/L	15 (48.4)	58 (50.4)	.840
≥14.5 U/L	16 (51.6)	57 (49.6)	
AST (%)			
<20.0 U/L	15 (48.4)	55 (47.8)	.956
≥20.0 U/L	16 (51.6)	60 (52.2)	
γ-GGT (%)			
<21.0 U/L	10 (32.3)	61 (53.0)	.040
≥21.0 U/L	21 (67.7)	54 (47.0)	
LDH (%)			
<250 U/L	25 (80.6)	99 (86.1)	.452
≥250 U/L	6 (19.4)	16 (13.9)	
TBIL (%)			
<12.10 μmol/L	18 (58.1)	54 (47.0)	.272
≥12.10 μmol/L	13 (41.9)	61 (53.0)	
DBIL (%)			
<2.72 μmol/L	18 (58.1)	55 (47.8)	.312
≥2.72 μmol/L	13 (41.9)	60 (52.2)	
IDBIL (%)	15 (48.4)	58 (50.4)	
<8.88 μmol/L	15 (48.4)	58 (50.4)	.840
≥8.88 μmol/L	16 (51.6)	57 (49.6)	
TP (%)			
<68.70 g/L	24 (77.4)	48 (41.7)	<.001
≥68.70 g/L	7 (22.6)	67 (58.3)	
ALB (%)			
<38.95 g/L	28 (90.3)	45 (39.1)	<.001
≥38.95 g/L	3 (9.7)	70 (60.9)	
GLOB (%)			
<29.10 g/L	18 (58.1)	55 (47.8)	.312
≥29.10 g/L	13 (41.9)	60 (52.2)	
A/G (%)			
<1.3	15 (48.4)	39 (33.9)	.138
≥1.3	16 (51.6)	76 (66.1)	
PALB (%)			
<200 mg/L	25 (80.6)	47 (40.9)	<.001
≥200 mg/L	6 (19.4)	68 (59.1)	
TRIG (%)			
<1.08 mmol/L	13 (41.9)	59 (51.3)	.354
≥1.08 mmol/L	18 (58.1)	56 (48.7)	
ALP (%)			
<89.50 U/L	15 (48.4)	58 (50.4)	.840
≥89.50 U/L	16 (51.6)	55 (49.6)	
Glu (%)			
<5.10 mmol/L	19 (61.3)	50 (43.5)	.078
≥5.10 mmol/L	12 (38.7)	65 (56.5)	
R (%)			
<4.34 10^9^/L	22 (71.0)	50 (43.5)	.007
≥4.34 10^9^/L	9 (29.0)	65 (56.5)	
Hct (%)			
<38.10 L/L	23 (74.2)	49 (42.6)	.002
≥38.10 L/L	8 (25.8)	66 (57.4)	
Hb (%)			
<122.50 g/L	23 (74.2)	50 (43.5)	.002
≥122.50 g/L	8 (25.8)	65 (56.5)	
CEA (%)			
<2.54 ng/mL	14 (45.2)	59 (51.3)	.544
≥2.54 ng/mL	17 (54.8)	56 (48.7)	
AFP (%)			
<3.02 ng/mL	21 (67.7)	52 (45.2)	.026
≥3.02 ng/mL	10 (32.3)	63 (54.8)	
CA199 (%)			
<14.40 U/mL	11 (35.5)	61 (53.0)	.083
≥14.40 U/mL	20 (64.5)	54 (47.0)	
CA724 (%)			
<2.56 U/mL	13 (41.9)	60 (52.2)	.312
≥2.56 U/mL	18 (58.1)	55 (47.8)	
CA125II			
<27.59 U/mL	9 (29.0)	64 (55.7)	.009
≥27.59 U/mL	22 (71.0)	51 (44.3)	

A/G = albumin/globulin, AFP = alpha-fetoprotein, ALB = albumin, ALT = alanine transaminase, APL = alkaline phosphatase, AST = Aspartate aminotransferase, CA125II = carbohydrate antigen 125II, CA199 = carbohydrate antigen 199, CA724 = carbohydrate antigen 724, CEA = carcinoembryonic antigen, DBIL = direct bilirubin, GLOB = globulin, Glu = glucose, GNRI = Geriatric Nutritional Risk Index, Hb = hemoglobin, Hct = hematocrit, IDBIL = Indirect bilirubin, LDH = lactate dehydrogenase, PALB = prealbumin, R = red blood cell, TBIL = total bilirubin, TP = total protein, IITRIG = triglyceride, γ-GGT = γ-glutamyl transferase.

### 3.2. Univariate and multivariate analysis

We observed that PALB, IDBIL, CEA, CA199, CA724, GNRI, radical resection, surgery, Borrmann type, TNM stage, Lauren type, treatment, PD-1, and PD-L1 were significantly associated with prognosis for both PFS and OS (all *P < *.05). Additionally, TBIL (*P* = .035) was found to be a prognostic factor for OS. Among them, CA724, GNRI, and TNM stage were identified as independent prognostic factors in this study (Table 3).

**Table 3:** Univariate and multivariate analysis for PFS and OS.

Parameters	PFS				OS			
	Univariate analysis		Multivariate analysis		Univariate analysis		Multivariate analysis	
	HR (95% CI)	*P*	HR (95% CI)	*P*	HR (95% CI)	*P*	HR (95% CI)	*P*
Sex								
Male	Ref				Ref			
Female	1.099 (0.645–1.872)	.729			1.084 (0.636–1.846)	.768		
Age	1.001 (0.975–1.028)	.931			0.998 (0.972–1.024)	.885		
BMI	0.976 (0.908–1.049)	.506			0.966 (0.896–1.041)	.358		
ALB	0.957 (0.903–1.014)	.136			0.946 (0.894–1.001)	.055		
GNRI								
≥92.00	Ref				Ref			
<92.00	2.430 (1.405–4.202)	.001	2.542 (1.292–5.000)	.007	2.421 (1.403–4.179)	.001	2.222 (1.208–4.086)	.01
TBIL								
<12.10 μmol/L	Ref				Ref			
≥12.10 μmol/L	1.742 (1.041–2.913)	.035	2.359 (0.819–6.800)	.112	1.601 (0.960–2.671	.071		
DBIL								
<2.72 μmol/L	Ref				Ref			
≥2.72 μmol/L	1.172 (0.707–1.944)	.538			1.129 (0.679–1.877)	.64		
IDBIL								
<8.88 μmol/L	Ref				Ref			
≥8.88 μmol/L	1.710 (1.026–2.851)	.04	1.339 (0.469–3.823)	.585	1.772 (1.065–2.949)	.028	1.750 (0.953–3.212)	.071
TP								
<68.70 g/L	Ref				Ref			
≥68.70 g/L	0.842 (0.511–1.386)	.498			0.789 (0.478–1.299)	.351		
PALB								
<200 mg/L	Ref				Ref			
≥200 mg/L	0.523 (0.313–0.872)	.013	0.749 (0.413–1.365)	.339	0.485 (0.291–0.811)	.006	0.656 (0.373–1.152)	.142
CEA								
<2.54 ng/mL	Ref				Ref			
≥2.54 ng/mL	1.725 (1.034–2.877)	.037	1.137 (0.604–2.139)	.691	2.154 (1.282–3.618)	.004	1.373 (0.736–2.562)	.32
AFP								
<3.02 ng/mL	Ref				Ref			
≥3.02 ng/mL	1.250 (0.758–2.061)	.382			1.184 (0.718–1.953)	.508		
CA199								
<14.40 U/mL	Ref				Ref			
≥14.40 U/mL	1.752 (1.050–2.923)	.032	1.093 (0.640–1.867)	.746	1.973 (1.181–3.298)	.009	1.055 (0.611–1.821)	.849
CA724								
<2.56 U/mL	Ref				Ref			
≥2.56 U/mL	2.245 (1.326–3.802)	.003	1.910 (1.079–3.381)	.007	2.370 (1.398–4.018)	.001	1.975 (1.111–3.513	.02
CA125II								
<27.59 U/mL	Ref				Ref			
≥27.59 U/mL	1.237 (0.751–2.039)	.404			1.168 (0.709–1.924)	.542		
Radical resection								
Yes	Ref				Ref			
No	2.501 (1.478–4.231)	.001	1.647 (0.642–4.227)	.299	2.800 (1.631–4.807)	0	2.209 (0.905–5.391)	.082
Surgery								
Yes	Ref				Ref			
No	2.069 (1.242–3.446)	.005	1.004 (0.441–2.287)	.993	2.093 (1.242–3.526)	.006	1.735 (0.739–4.073)	.206
Primary tumor site								
Low 1/3	Ref				Ref			
Others*	0.944 (0.573–1.555)	.822			0.985 (0.598–1.622)	.952		
Borrmann type								
I + II	Ref				Ref			
III + IV + Unknown	1.625 (1.023–2.583)	.04	1.291 (0.437–3.817)	.644	1.636 (1.023–2.616)	.04	1.416 (0.505–3.973)	.508
Tumor size								
<50 mm	Ref				Ref			
≥50 mm + Unknown	1.131 (0.847–1.511)	.405			1.140 (0.853–1.523)	.377		
Differentiation								
Poor	Ref				Ref			
Others	1.131 (0.847–1.511)	.405			1.140 (0.853–1.523)	.377		
Pathology								
Adenocarcinoma	Ref				Ref			
Others	1.386 (0.947–2.028)	.093			1.380 (0.947–2.011)	.094		
TNM stage								
I	Ref				Ref			
II	1.431 (0.855–2.395)	.172	1.899 (1.039–3.469)	.037	1.430 (0.856–2.387)	.172	1.817 (0.999–3.304)	.05
III	2.134 (1.269–3.591)	.004	2.343 (1.275–4.303)	.006	2.207 (1.319–3.694)	.003	2.465 (1.349–4.503)	.003
IV	3.837 (2.299–6.405)	<.001	3.615 (2.003–6.524)	<.001	3.161 (1.909–5.234)	<.001	2.928 (1.636–5.239)	<.001
Lauren type								
Intestinal	Ref				Ref			
Others	1.415 (1.125–1.781)	.003	1.091 (0.758–1.568)	.64	1.426 (1.133–1.796)	.002	1.067 (0.745–1.530)	.723
Treatment								
Chemotherapy	Ref				Ref			
ICIs	2.812 (1.558–5.074)	.001	1.380 (0.645–2.956)	.407	3.078 (1.696–5.586)	0	1.452 (0.686–3.074)	.33
PD-1								
Positive	Ref				Ref			
Negative + Unknown	1.602 (1.205–2.129)	.001	2.236 (0.493–10.139)	.297	1.694 (1.272–2.256)	0	2.917 (0.621–13.704)	.175
PD-L1								
Positive	Ref				Ref			
Negative + Unknown	1.747 (1.257–2.430)	.001	1.032 (0.433–2.459)	.943	1.863 (1.336–2.597)	0	1.087 (0.475–2.488)	.844

AFP = alpha-fetoprotein, ALB = albumin, BMI = body mass index, CA125II = carbohydrate antigen 125II, CA199 = carbohydrate antigen 199, CA724: = carbohydrate antigen 724, CEA = carcinoembryonic antigen, DBIL = direct bilirubin, GNRI = Geriatric Nutritional Risk Index, ICIs = immune checkpoint inhibitors, IDBIL = Indirect bilirubin, PALB = prealbumin, TBIL = total bilirubin, TP = total protein.

* Others of Primary tumor site was upper 1/3 + Middle 1/3 + whole; Others of Pathology was mucinous carcinoma + signet ring cell carcinoma + mixed carcinoma + unknown; Others of Differentiation was Moderately + Well + Unknown; Others of Lauren type was Diffuse + Mixed + Unknown.

### 3.3. Survival analysis

**Figure 1.**
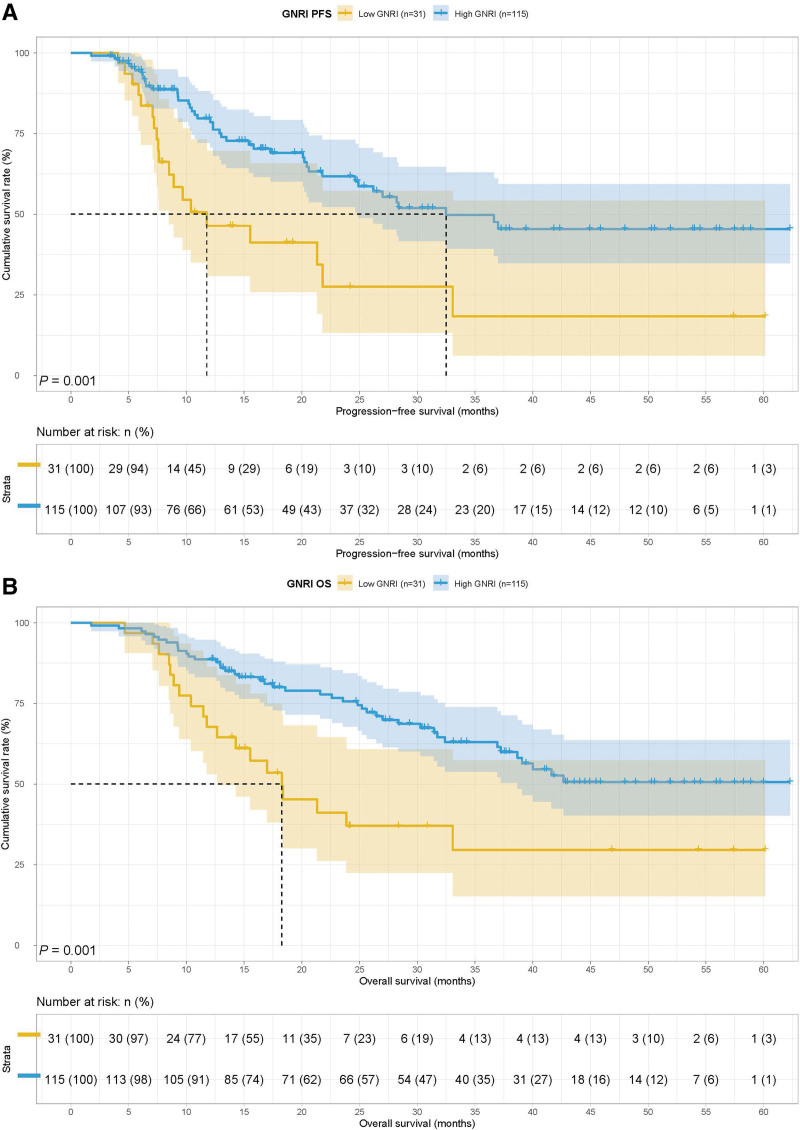
Geriatric nutritional risk index related survival curve of (A) PFS and (B) OS. OS = overall survival, PFS = progression-free survival.

**Figure 2.**
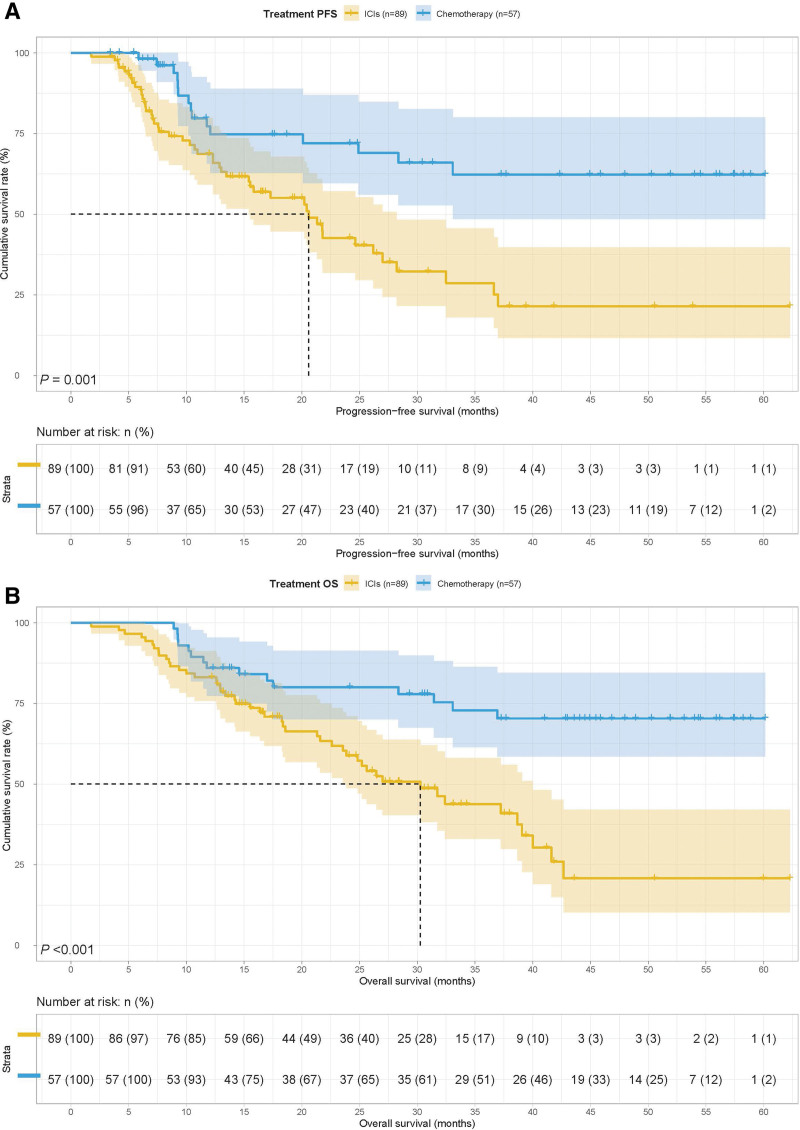
Treatment related survival curve of (A) PFS and (B) OS. OS = overall survival, PFS = progression-free survival.

In all cases, patients with GNRI < 92.00 had poorer PFS (21.33 months vs 28.37 months, *P* = .001) and OS (33.06 months vs 41.63 months, *P* = .001) (Fig. 1A and B). We conducted separate analyses for patients who received ICIs (n = 89) and chemotherapy (n = 57). Correlation analysis showed that treatment was related to surgery, tumor size, TNM stage, and Lauren type (all *P* < .05). In addition, patients receiving ICIs were more likely to have an unknown Borrmann type by Fisher’s exact test (Table 4). Patients in the ICIs group had poorer PFS (20.60 months vs not reached, *P* = .001) and OS (30.27 months vs not reached, *P* < .001) (Fig. 2A and B).

**Table 4:** The clinical information of different treatment.

n	ICIs	Chemotherapy	*P*
	89	57	
Age (%)	58.39 (10.26)	57.34 (9.29)	.537
Surgery (%)			
Yes	38 (42.7)	48 (84.2)	<.001
No	51 (57.3)	9 (15.8)	
Borrmann type (%)			
I + II	6 (6.8)	3 (5.4)	<.001
III + IV	32 (35.2)	45 (78.6)	
Unknown	51 (58.0)	9 (16.1)	
Tumor size (%)			
<50 mm	19 (21.3)	22 (38.6)	.046
≥50 mm	19 (21.3)	13 (22.8)	
Unknown	51 (57.3)	22 (38.6)	
TNM stage (%)			
I	3 (3.4)	7 (12.5)	<.001
II	5 (5.7)	9 (16.1)	
III	14 (15.9)	24 (42.9)	
IV	66 (75.0)	16 (28.6)	
Lauren type (%)	14 (15.7)	19 (33.3)	
Intestinal	14 (15.7)	19 (33.3)	<.001
Diffuse	8 (9.0)	13 (22.8)	
Mixed	8 (9.0)	15 (26.3)	
Unknown	59 (66.3)	10 (17.5)	

**Figure 3.**
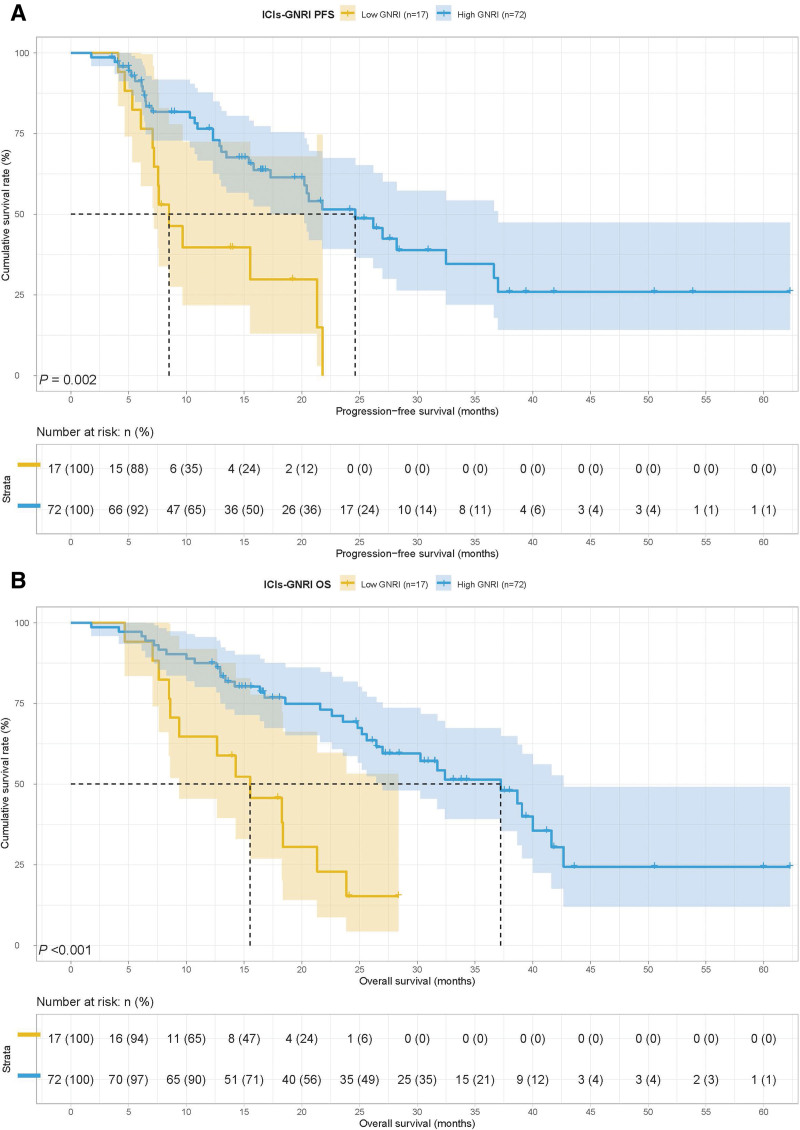
Geriatric nutritional risk index related survival curve of (A) PFS and (B) OS in patients with ICIs. ICIs = immune checkpoint inhibitors, OS = overall survival, PFS = progression-free survival.

**Figure 4.**
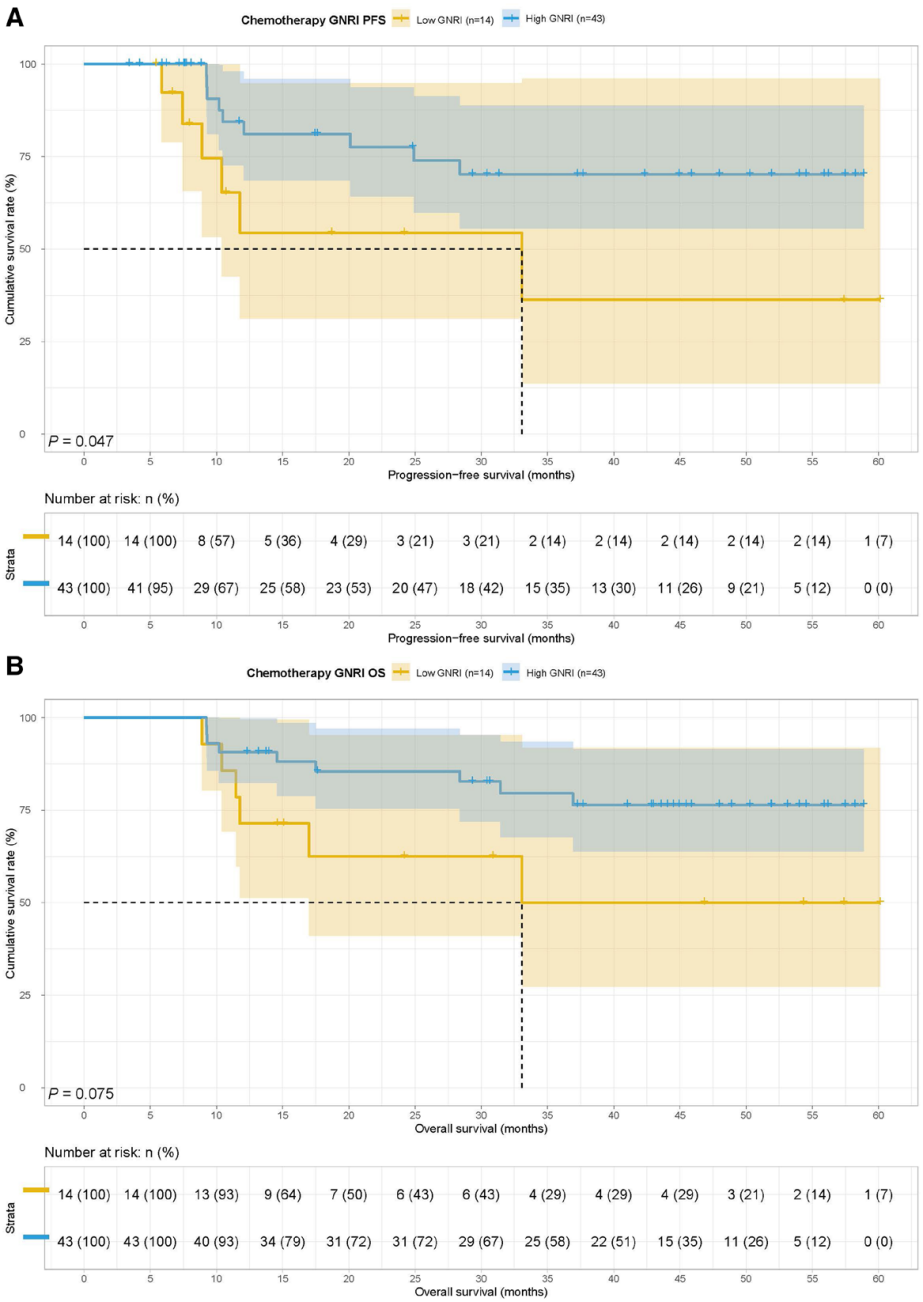
Geriatric nutritional risk index related survival curve of (A) PFS and (B) OS in patients with chemotherapy. OS = overall survival, PFS = progression-free survival.

Among the ICIs group, 17 cases had GNRI < 92.00 and 72 cases had GNRI ≥ 92.00. The low GNRI group had poorer PFS (8.50 months vs 24.63 months, *P* = .002) and OS (15.53 months vs 37.23 months, *P* < .001) (Fig. 3A and B). Among the chemotherapy group, 14 cases had GNRI < 92.00 and 43 cases had GNRI ≥ 92.00. The median survival time (MST) for PFS and OS in the low GNRI group was 33.07 months. However, the MST for PFS and OS in the high GNRI group were both not reached. Patients with GNRI < 92.00 had a shorter PFS (33.07 months vs not reached, *P* = .047) and OS (33.07 months vs not reached, *P* = .075) (Fig. 4A and B).

### 3.4. Nomograms to predict survival probability

**Figure 5.**
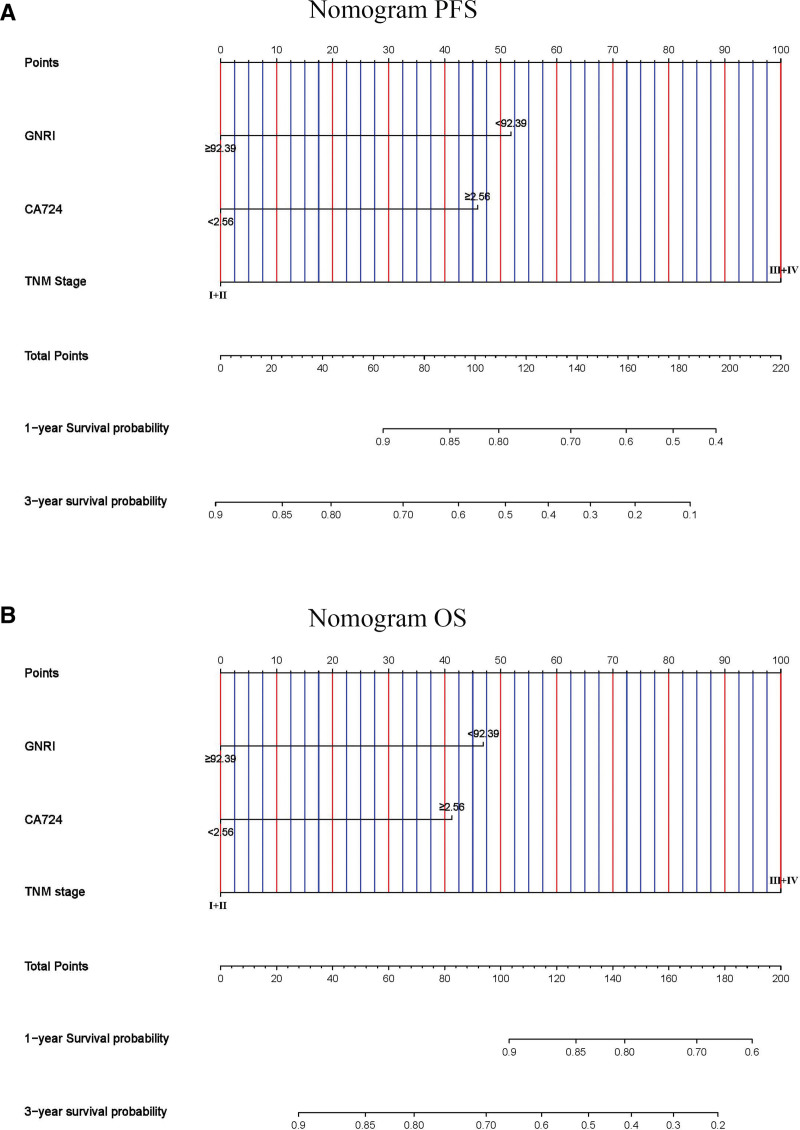
Nomograms for predicting 1- and 3-year survival probability of (A) PFS and (B) OS. OS = overall survival, PFS = progression-free survival.

**Figure 6.**
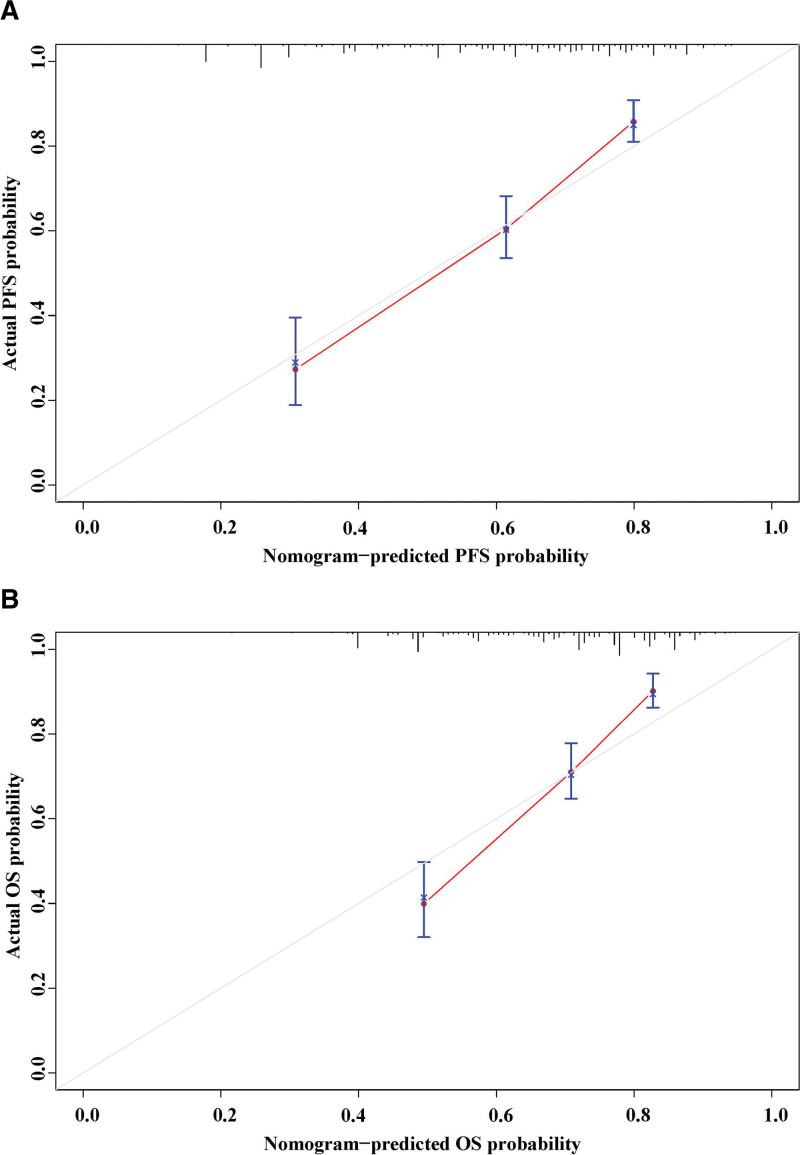
The calibration curves of the nomograms for PFS (A) and OS (B). OS = overall survival, PFS = progression-free survival.

We developed nomograms (Fig. 5A and B) to predict the survival probability, and the C-index for these nomograms was 0.667 (0.600–0.735) and 0.685 (0.622–0.749) for PFS and OS, respectively. We also performed bootstrap correction for the nomograms and drew calibration curves, which demonstrated their high predictive accuracy (Fig. 6A and B).

## 4. Discussion

The factors that influence the efficacy of immunotherapy in gastric cancer patients are complex and diverse. Although previous studies have demonstrated the significant therapeutic benefits of ICIs, not all patients respond to immunotherapy, and most do not derive any benefits.^[22,24–26]^ Moreover, the existing biomarkers for predicting the efficacy of immunotherapy are still not entirely accurate.^[27]^ Several inflammations or nutrition-based scores, utilizing clinical markers, have been shown to be capable of predicting the prognosis of gastric cancer patients receiving immunotherapy.^[28]^ Therefore, the continued investigation of GNRI’s ability to predict the effectiveness of immunotherapy is of great importance, given that it is a simple and reliable nutritional and prognostic marker.

The original purpose of GNRI was to foresee the nutritional risk of elderly hospitalized patients, low GNRI group patients were more prone to nutritional risk.^[19]^ The follow-up studies also obtained similar results.^[29,30]^ The prognosis of individuals with multiple cancers could be predicted by GNRI. Karayama et al examined the relationship between the survival rate and GNRI in lung cancer patients and discovered that low GNRI had substantially shorter survival times than patients with high GNRI.^[23]^ Between 2002 and 2013, Nakayama et al gathered 248 patients with advanced head and neck cancer and discovered that GNRI was a useful predictive factor in those with advanced head and neck cancer.^[31]^ In addition, GNRI was applied to gastrointestinal cancer. Wang et al assessed and contrasted the use of PNI, NRI, GNRI, and CONUT in patients with esophageal cancer. They collected 192 patients with esophageal cancer for analysis and found that GNRI was the best predictor of perioperative morbidities.^[20]^ Matsunaga et al collected 497 gastric cancer patients with surgery in 12 institutions and compared the predictive ability for disease-specific survival (DSS) and OS of GNRI, PNI, NLR, and PLR with the ROC curve. The result showed that GNRI had the best predictive ability for DSS and OS.^[21]^ Sugawara et al compared the predictive ability for OS of GNRI, nutrition indices, and systemic inflammatory markers in 1166 gastric cancer patients. GNRI also had a superior predictive ability for OS.^[32]^ Moreover, GNRI was linked to the prognosis of older cancer cachexia patients. Low GNRI was discovered to be an independent predictive factor for the poor outcome of cancer cachexia patients in multi-center cohort research.^[33]^ In a word, GNRI is a great predictor of gastric cancer.

This study investigated the prognostic predictive value of GNRI in patients with gastric cancer treated with ICIs or chemotherapy. The result displayed that patients with GNRI < 92.00 had shorter PFS and OS in all patients. The subgroup analysis obtained similar results, patients with low GNRI were significantly associated with poorer PFS and OS, especially in patients with ICIs group. Moreover, GNRI, CA724, and TNM stage were identified as independent predictive factors for both PFS and OS. The nomograms created by the outcomes of the multivariate analysis have likewise demonstrated excellent predictability for PFS and OS survival outcomes.

In survival analysis, we found that patients with ICIs had shorter survival times. This result seems to be surprising, but the current application strategy of immunotherapy in gastric cancer patients can explain this phenomenon. In our institution, most patients are considered for ICIs only if they are unable to undergo radical resection or are chemotherapy resistant, and they are often more symptomatic or have distant metastases, resulting in shorter PFS and OS. Correlation analysis also support this explanation, patients who received ICIs have higher TNM stage, more non-radical surgery, and more unknown pathology information.

GNRI contains serum albumin and body weight, which are both significant prognostic factors for cancer. Previous studies have shown that albumin was a biomarker for systemic inflammation, and hypoalbuminemia indicated that patients were in a state of systemic inflammation.^[34]^ Systemic inflammation is not only the main cause of malnutrition but also an important factor leading to tumor progression.^[35–38]^ In the inflammatory state, the synthesis of albumin is suppressed by some cytokines.^[39]^ At the same time, albumin degeneration occurred to oxidative stress.^[40]^ Hypoalbuminemia can also lead to decreased immune function thus leading to tumor progression. The loss of body weight reflected poor nutritional status and insufficient energy reserves, and malnutrition affected normal immune function.^[41]^ Several studies have also found that BMI was related to the efficacy of ICIs. Patients with high BMI had a longer survival time after receiving ICIs.^[42,43]^ The efficacy of ICIs depends on human normal immunity function. Both albumin and body weight can affect the patient’s immune function, thus affecting the therapeutic effect of ICIs. Therefore, GNRI combined with serum albumin and body weight can effectively predict the efficacy of ICIs.

There were still some unsolvable problems in this study. First, this was a single center retrospective study with potential information bias. Second, the effect of different immune checkpoint inhibitors on patients was not considered in this study. Third, GNRI could combine with some other indicators such as C-reactive protein to increase its predictive ability for the clinical outcomes of patients. This study’s results need to be supported by larger sample sizes and better designed randomized controlled trials.

## 5. Conclusion

GNRI was significantly associated with survival time in patients with gastric cancer who received ICIs, patients with low GNRI had shorter PFS and OS. GNRI might be able to identify patients who might benefit from ICIs.

## Author contributions

**Conceptualization:** Limin Zhang.

**Project administration:** Limin Zhang.

**Resources:** Limin Zhang.

**Writing – original draft:** Bao Liu.

**Writing – review & editing:** Bao Liu.
